# ISG15, a Small Molecule with Huge Implications: Regulation of Mitochondrial Homeostasis

**DOI:** 10.3390/v10110629

**Published:** 2018-11-13

**Authors:** Manuel Albert, Martina Bécares, Michela Falqui, Carlos Fernández-Lozano, Susana Guerra

**Affiliations:** Department of Preventive Medicine, Public Health and Microbiology, Universidad Autónoma, E-28029 Madrid, Spain; manuel.albert@uam.es (M.A.); martina.becares@uam.es (M.B.); michela.falqui@uam.es (M.F.); carlos.fernandezl@uam.es (C.F.-L.)

**Keywords:** interferon, ubiquitin-like modification, mitochondria, mitophagy, OXPHOS

## Abstract

Viruses are responsible for the majority of infectious diseases, from the common cold to HIV/AIDS or hemorrhagic fevers, the latter with devastating effects on the human population. Accordingly, the development of efficient antiviral therapies is a major goal and a challenge for the scientific community, as we are still far from understanding the molecular mechanisms that operate after virus infection. Interferon-stimulated gene 15 (ISG15) plays an important antiviral role during viral infection. ISG15 catalyzes a ubiquitin-like post-translational modification termed ISGylation, involving the conjugation of ISG15 molecules to *de novo* synthesized viral or cellular proteins, which regulates their stability and function. Numerous biomedically relevant viruses are targets of ISG15, as well as proteins involved in antiviral immunity. Beyond their role as cellular powerhouses, mitochondria are multifunctional organelles that act as signaling hubs in antiviral responses. In this review, we give an overview of the biological consequences of ISGylation for virus infection and host defense. We also compare several published proteomic studies to identify and classify potential mitochondrial ISGylation targets. Finally, based on our recent observations, we discuss the essential functions of mitochondria in the antiviral response and examine the role of ISG15 in the regulation of mitochondrial processes, specifically OXPHOS and mitophagy.

## 1. Introduction

### 1.1. ISG15 Definition

The innate immune response is the first line of defense against microbial and viral infections. Invading microorganisms produce danger- and pathogen-associated molecular patterns that interact with host pattern-recognition receptors, triggering several intracellular signaling cascades that activate nuclear factor *kappa*-B (NF-κB), mitogen-activated protein kinases (MAPKs) and interferon (IFN) regulatory factors (IRFs), resulting in the expression of a broad array of proteins involved in host defense such as type-I IFNs and proinflammatory cytokines [1,2]. The release of type-I IFNs has both autocrine and paracrine effects *via* IFNα/β receptors (IFNARs) on the cell surface. Binding to IFNARs leads to the activation of the Janus kinase-signal transducer and activator of transcription proteins (JAK-STAT) signaling pathway and the formation of the interferon-stimulated gene factor 3 (ISGF3) complex, with the subsequent expression of IFN-stimulated genes [3] that establish an antiviral state and play important roles in determining the host innate and adaptive immune responses [4].

**Figure 1:**
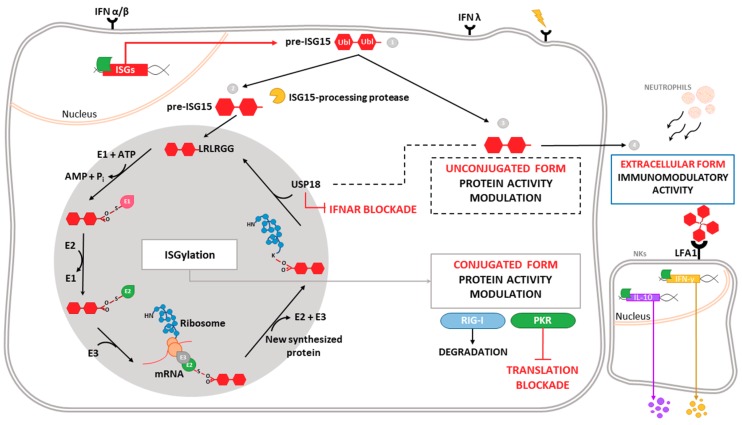
*Intracellular and extracellular activities of ISG15.* Different stimuli trigger the expression of ISG15, which is produced as a precursor of 17 kDa with two ubiquitin-like domains linked by a hinge region (1). Intracellular ISG15 can be processed into its mature form and conjugated to *de novo* synthesized proteins in a process termed ISGylation. ISG15 processing exposes its carboxy-terminal LRLRGG motif, allowing its conjugation to lysine residues in target proteins to modulate their function. In addition, ISGylation is reversible due to the action of the protease USP18, which also regulates IFNAR-mediated signaling (2). ISG15 can remain unconjugated within the cell, regulating protein activity (3), or be secreted as a cytokine, acting as a chemotactic and stimulating factor for immune cells (4). Binding of ISG15 to LFA-1 integrin receptor on the surface of NK cells promotes the activation, production and release of IFN-γ IL-10 after IL-12 priming. Moreover, extracellular ISG15 is able to form dimers/multimers through cysteine residues, to modulate cytokine levels.

One of the most highly induced genes in the type-I IFN signaling cascade is *ISG15* (interferon-stimulated gene 15), which encodes a small ubiquitin-like protein involved in a post-translational modification (PTM) process termed ISGylation. Through this process, ISG15 covalently binds to a wide range of target proteins [5]. ISG15 exists in three different forms: unconjugated within the cell, conjugated to target proteins, and released into the serum (Figure 1). ISG15 is synthesized as a 17-kDa precursor that is proteolytically processed into a mature form of 15 kDa. This processing exposes a carboxy-terminal LRLRGG motif, required for ISGylation [6] (Figure 1). ISGylation is the result of the coordination of three enzymatic activities-activation, conjugation and ligation—performed by ISG15-activating enzymes (E1), ISG15-conjugating enzymes (E2) and ISG15-ligating enzymes (E3), respectively [7] (Figure 1). Considering the broad substrate selectivity described for ISGylation, and the fact that Herc5 (the major ISG15-ligating enzyme) associates with polyribosomes, it has been established that ISGylation targets proteins undergoing active translation [8]. In the context of viral infection, those newly synthesized proteins are largely viral proteins and cellular proteins involved in the innate immune response.

ISG15 conjugation to target proteins is a covalent and reversible process through the action of a 43-kDa deISGylase enzyme, ubiquitin-specific protease 18 (USP18) [9,10]. Interestingly, both ISG15 and its conjugating and deconjugating enzymes are upregulated by type-I IFN [9], as well as by other stimuli such as type-II and type-III IFNs [11,12,13], lipopolysaccharide [14], retinoic acid [15], DNA damage or genotoxic reagents [16]. USP18 not only acts as a deconjugating enzyme, but also as a negative regulator of the type-I IFN pathway (Figure 1), with important implications in antiviral and antibacterial responses, immune cell development, autoimmune diseases and cancer [17]. In humans, ISG15 binds to USP18, increasing its stability and leading to a decrease in IFN-α/β signaling. Consequently, ISG15 deficiency results in low USP18 levels, and therefore a sustained elevation in ISG expression. This role for ISG15, which is absent in mice, seems to be predominant in humans, since patients appear not to be more susceptible to viral infections [18,19].

Beyond the above-mentioned forms of ISG15—conjugated to target proteins or unconjugated within the cell—ISG15 is also secreted into the serum, mainly by granulocytes *via* their secretory pathway [20]. Lymphocyte function-associated antigen 1 receptor (LFA1) has recently been identified as the cellular receptor for ISG15 (Figure 1). ISG15 binding to LFA1 triggers the activation of SRC family kinases, promoting IFN-γ and Interleukin-10 (IL-10) secretion in natural killer (NK) cells and, likely, also T-lymphocytes [21]. The role of ISG15 as an inductor of IFN-γ secretion seems to be the basis for the increased susceptibility to mycobacterial diseases in patients lacking a functional form of ISG15 [20]. Secreted ISG15 has also been described to promote NK [22] and dendritic cell [23] maturation, and to act as a chemotactic factor for neutrophils [24]. Along this line, a recent study highlighted the presence of dimeric and multimeric forms of extracellular ISG15 important for its cytokine activity during parasite infection, and speculated on the existence of an unknown ISG15 receptor on dendritic cells that mediates chemotaxis of these cells to the site of infection and IL-1β production [25]. 

Although there are several features of ISG15 that are shared with ubiquitin, specially its structure, conjugation and deconjugation mechanisms [26], ISGylation has not been shown to stimulate proteasomal degradation of its substrates [10]. Furthermore, some of the ISGylation consequences are exerted by restricting the ubiquitin system, what might be mediated through the conjugation of ISG15 to different E2 and E3 ubiquitin-conjugating enzymes [27], or even through the formation of mixed ubiquitin–ISG15 chains [28]. As a result, ISGylation can decrease the polyubiquitylated proteins levels and downregulate protein turnover by the proteasome system [28]. Additionally, unlike ubiquitin, no poly-ISG15 chains or specific ISG15-interacting motifs have been identified yet.

In the following sections, we discuss the antiviral mechanisms mediated by ISGylation of both viral and cellular proteins, with a focus on mitochondrial proteins, as we recently showed that ISG15 modulates essential mitochondrial metabolic processes such as respiration and mitophagy in macrophages, with important implications for innate immune responses [29].

### 1.2. Antiviral Role of ISG15 and ISGylation

The antiviral activity associated with ISG15 and/or ISGylation has been widely described since the first observation that *ISG15*-/- mice were more susceptible to viral infections than their wild-type counterparts, albeit the role of ISG15 and ISGylation in viral life cycles is specific to the virus involved [30]. Early studies using *ISG15*-/- mice demonstrated that ISG15 has a protective effect against lethal infection by Influenza virus, Herpes Simplex virus (HSV-1) and Sindbis virus (SINV) [31]. Similarly, mice deficient in UbE1l—the E1 enzyme of ISG15—were also more susceptible to lethal infection by SINV [32]. Moreover, exogenous expression of wild-type ISG15 by recombinant chimeric SINV protected IFNAR-/- mice against systemic and lethal infections, whereas expression of ISG15 mutants unable to conjugate to proteins did not show this protective effect [33], indicating an intrinsic antiviral role for ISGylation. It should be noted that such an antiviral effect could be due to the conjugation of ISG15 to viral and/or cellular proteins. By contrast, free ISG15, but not ISGylation, has been described to promote antiviral responses against Chikungunya virus (CHIKV) infection [34]. 

To date, an antiviral effect mediated by ISG15 or ISGylation has been described using in vitro and/or in vivo systems for many other DNA and RNA viruses, including Hepatitis B virus [35], Vesicular stomatitis virus [36,37], Respiratory syncytial virus [38,39], Human immunodeficiency virus type 1 (HIV-1) [40], and Ebola virus [27]. The antiviral effect of ISG15 and ISGylation has also been described against viruses of the genera Novirhabdovirus, Birnavirus and Iridovirus in zebrafish, an example of the evolutionary conservation of the antiviral role of ISG15 among vertebrates [41].

Given the importance of the antiviral response governed by ISG15, it is not surprising that viruses have evolved strategies to counteract its antiviral effects. For example, Influenza B virus (IBV) NS1 protein [42], Vaccinia virus E3 protein [43], and Human cytomegalovirus (HCMV) IE1 and PUL26 proteins obstruct ISG15 antiviral action by preventing ISGylation [44]. Similar mechanisms are also described for Orthonairovirus and Arterivirus OTU-domain-containing proteases [45] and for Coronavirus papain-like proteases (PLpro), which cleave ISG15 from target proteins. Remarkably, a PLpro inhibitor was shown to protect mice from lethal infection in vivo [46]. Surprisingly, it has been reported that ISGylation is necessary for robust production of Hepatitis C virus (HCV), conferring a novel role for ISG15 as a proviral factor that promotes virus production. Indeed, in human hepatocytes, siRNA silencing of ISG15 was sufficient to both inhibit HCV replication and increase IFN expression [47]. Several reports have now highlighted a role for ISG15 in the monitoring of HCV replication in cell cultures, as well as in the maintenance of HCV in liver, and pinpoint ISG15 as among the predictor genes for non-response to IFN therapy [48]. 

### 1.3. ISGylated Viral Proteins

Regarding the direct antiviral effect of ISGylation *via* conjugation to viral proteins, perhaps the best-known example is the Influenza A virus (IAV) NS1 protein. This non-structural protein is abundantly expressed in infected cells and acts in multiple stages of the viral cycle, with important roles in IFN antagonism including sequestering double-stranded RNA (dsRNA), inhibiting dsRNA-activated protein kinase (PKR) and contributing to the nuclear export of viral mRNAs while blocking the splicing and export of cellular mRNAs [49]. Seven lysine (K) residues in the NS1 protein were identified as potential target sites of ISGylation [50]. Specifically, ISG15 binding to K41, which is part of the NS1 nuclear-localization signal, prevents its interaction with importin-α, inhibiting the translocation of NS1 to the nucleus and therefore repressing IAV replication and viral RNA processing [51]. Moreover, ISGylation of the IAV NS1 protein blocks its ability to counteract the innate immune response, prevents its interaction with PKR and, therefore, restores IFN-induced antiviral activities against IAV [50].

Beyond NS1, Influenza virus nucleoprotein (NP) and matrix protein (M1) have also been reported as targets of ISG15 conjugation. ISGylated NP hinders the oligomerization of the more abundant unconjugated NP, acting as a dominant-negative inhibitor of NP oligomerization, impeding the formation of viral ribonucleoproteins and causing decreased viral protein synthesis and virus replication [52]. Interestingly, this study also identified a new role for Influenza B virus NS1 in the sequestration of ISGylated viral proteins, especially ISGylated NPs, which is perhaps an evolutionary mechanism to block the antiviral effect of ISGylation.

Another example of ISGylation of a viral protein with antiviral effects is the 2A protease (2Apro) of Coxsackievirus B3 (CVB3). ISG15 conjugation to 2Apro inhibits its ability to cleave the eukaryotic initiation factor eIF4G in cardiomyocytes, hindering the translational shutoff induced by CVB3 infection [53]. Consequently, ISG15 conjugation to CVB3 leads to a reduction in virus titers and limits inflammatory cardiomyopathy, heart failure and lethality [53]. Similarly, ISGylation of the HCMV scaffold protein pUL26 interferes with the viral modulation of the innate immune response. Specifically, ISGylation of pUL26 at K136 and K169 inactivates its function in the downregulation of TNFα-mediated NF-κB activation, suppressing HCMV growth [44]. Finally, another example of an ISGylated viral protein is the Human papillomavirus (HPV) L1 capsid protein. ISGylated L1 proteins were shown to be incorporated into HPV pseudoviruses, resulting in a reduced infectivity; the precise mechanism that mediates this inhibitory effect remains elusive [8].

### 1.4. ISGylated Cellular Proteins

Knowledge about the impact of host protein ISGylation in virus replication and cell homeostasis is still scant. In contrast to ubiquitylation, the molecular effect of ISG15 conjugation on target proteins is not always clear. Protein ISGylation has been reported to increase protein degradation by selective autophagy [54], but there are also many examples where ISGylation inhibits ubiquitylation, frustrating proteasome-mediated degradation of target proteins [55,56,57]. 

With regard to proteins involved in antiviral response, many effectors of IFN signaling such as PKR [58], retinoic acid-inducible gene-I (RIG-I) [59] and *Myxoma* resistance protein 1 (MxA) [60] have been reported to be targets of ISGylation. PKR ISGylation at K69 and K159, both located in the dsRNA-binding motif, triggers its activation. This modification occurs in the absence of viral RNA and leads to the phosphorylation of eIF2α, preventing protein translation [58] and suggesting that ISGylation might mediate the activation of PKR in response to stressful stimuli beyond viral infection. Further, ISG15 conjugation to RIG-I decreases RIG-I cellular levels and downregulates RIG-I-mediated signaling. Accordingly, ISGylation of RIG-I represents a negative feedback loop that might control the strength of the antiviral response [59]. Interestingly, free ISG15 also regulates RIG-I levels by promoting the interaction between RIG-I and the autophagic cargo receptor p62, mediating RIG-I degradation *via* selective autophagy [61]. The interferon-induced MxA protein is also a target of ISGylation, though the effect of this modification is not clear.

Other proteins involved type-I IFN signaling and regulation, such as components of the JAK-STAT pathway or regulators of signal transduction (e.g., JAK1 and extracellular signal-regulated kinase 1 [ERK1]), are also bound by ISG15, although the functional consequences of ISGylation remain unknown [9,62]. Moreover, interferon regulatory factor 3 (IRF3), STAT1 and the actin-binding protein Filamin B are also targets for ISG15 conjugation, with implications in the development of the innate immune response. IRF3 is ISGylated at K193, K360 and K366, which attenuates its interaction with the peptidyl-prolyl isomerase PIN1, preventing IRF3 ubiquitylation. Thus, ISGylation of IRF3 sustains its activation and enhances IRF3-mediated antiviral responses by inhibiting its degradation [63]. In a similar manner, ISGylation of phosphorylated STAT1 (pSTAT1) inhibits its polyubiquitylation and further proteasomal degradation, supporting sustained STAT1 activation [57]. ISGylation of Filamin B, which acts as a scaffold of IFN signaling mediators, negatively regulates IFNα-induced c-Jun N-terminal kinases (JNK) signaling, preventing apoptosis induction [64].

Beyond antiviral response, ISGylation has been described to block the process of virus budding by interfering with the endosomal sorting complexes required for transport (ESCRT) machinery. For example, ISGylation of CHMP5 triggers its aggregation and the sequestration of the Vps4 cofactor LIP5, impairing the membrane recruitment of Vps4 and its interaction with the Gag budding complex of Avian sarcoma leukosis virus and HIV-1, leading to the inhibition of virus release from the cell [65]. Similarly, ISGylation of tumor susceptibility gene 101 protein (TSG101), another component of the ESCRT sorting complex, inhibits the trafficking of viral hemagglutinin to the cell surface during IAV infection [66], blocking virus release. ISG15 has also been described to inhibit the interaction of HIV-1 Gag protein with TSG101, underscoring a critical role of ISG15 in the IFN-mediated inhibition of HIV-1 budding and release [40]. This sorting mechanism is also used in the generation of exosomes, which are small vesicles secreted to the extracellular environment by most cell types. Interestingly, ISGylation of TSG101 has been recently reported to inhibit exosome secretion [67].

The above examples serve to illustrate the relevance of ISGylation in the induction and regulation of the antiviral response (for a more complete review of ISGylated cellular proteins see Reference [30]), and highlight the complexity of fully understanding the consequences of ISGylation in the regulation of biochemical processes where it is involved. Although the significance of ISGylation of host proteins has been elucidated for only a small set of cellular proteins, ISGylation has a broad target specificity, and there is increasing evidence for its role in regulating many cellular functions. To address this concept, several proteomic studies have been performed to determine ISGylated host proteins. Zhao et al. [60] transfected a tagged ISG15 protein into IFN-stimulated HeLa cells, and used affinity selection to identify 158 ISGylated proteins. In a similar approach, Giannakopoulos et al. [68] used IFN-stimulated USP18-/- mouse embryonic fibroblasts and human U937 cells to detect up to 76 proteins conjugated to endogenously-expressed ISG15. A third proteomic study [69] identified 174 ISGylated cellular proteins in IFN-stimulated A549 human lung adenocarcinoma cells stably expressing FLAG-ISG15. More recently, Peng et al. [70] examined ISGylated proteins in Influenza virus-infected A549 cells, identifying a total of 22 cellular proteins in addition to viral NS1 protein. We have surveyed the proteins identified by these four studies, which rendered up to 330 cellular proteins. Identified proteins include, as previously outlined [8], abundant constitutively expressed proteins as well as diverse interferon-induced proteins. Interestingly, there is only a low degree of overlap between the studies, and only four proteins are common to all four analyses (the glycolytic enzymes ALDO1 and ENO1, the peroxiredoxin PRDX1, and STAT1). These discrepancies may reflect the different transcriptional/translational patterns of the different cell lines included in each study, as it is believed that the biological effects of ISGylation are dynamic and cell type/tissue-specific [5].

**Figure 2:**
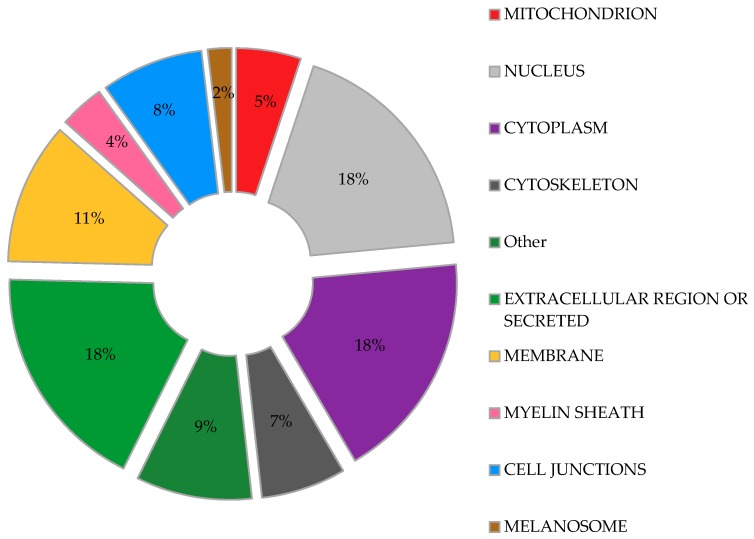
*Predicted subcellular distribution of ISGylated proteins.* Proteins identified as ISGylation targets in different proteomic studies were evaluated for their subcellular location. Percentage of the total ISGylated proteins located in each cellular organelle is shown.

We used DAVID bioinformatics resources [71,72] to determine the subcellular localization of the proteins identified as ISGylation targets in the aforementioned studies, with the aim to obtain a comprehensive picture of the broad range of actions of ISG15. In agreement with a previous report [73], our analysis (Figure 2) shows that ISG15-targeted proteins are found almost throughout the cell, including nucleus, perinuclear space, cytosol, mitochondria, rough endoplasmic reticulum and cell membranes [73]. Moreover, a similar percentage of ISGylation targets were predicted to be located in the nucleus, cytoplasm, extracellular space or as secreted proteins (Figure 2). Interestingly, proteins associated with cytoskeleton and cell junctions represent a significant percentage of the ISG15 target proteins. Other cell structures such as the melanosome or myelin sheath were also represented in the study, perhaps accounting for a specific role of ISGylation in these organelles.

The potential role of ISG15 in mitochondria seems to be relevant, as a recent study predicted that 17% of free ISG15 was localized to mitochondria [13]. In our own analysis of the above proteomic studies, fifty-two ISGylated proteins were predicted to localize to mitochondria, representing about 5% of the total ISG15 target proteins (Figure 2). Further examination of these potentially ISGylated proteins indicate that different mitochondrial processes could be affected by ISG15 conjugation (Table 1). Remarkably, several subunits of the ATP synthase (complex V of the respiratory chain) appear to be ISG15 targets, which may be of relevance as mitochondrial ATP production is the main source of energy for the cell. In line with these observations, our recent work linked ISG15 to the control of the mitochondrial oxidative metabolism in macrophages in the context of viral infection [29]. Based on the evident association between ISG15 and mitochondria, we will briefly review the role of mitochondria as antiviral mediators and targets of ubiquitin-like modifiers, focusing on the current knowledge about ISG15- and ISGylation-mediated regulation of these multifunctional organelles. 

## 2. Mitochondria: Key Organelles in Antiviral Responses

Mitochondria have myriad functions in the cell although they are best known for providing energy in the form of ATP and for controlling metabolism to maintain energy homeostasis. Owing to their endosymbiotic origin, mitochondria have their own genome, a single 16-kb circular DNA which codes for 13mitochondrial proteins, 2 ribosomal RNAs and 22 transfer RNAs [74]. The remainder of mitochondrial proteins are encoded by nuclear DNA and are then transported to the mitochondria through the recognition of amino acid sequences known as mitochondrial targeting signals [75]. As double-membrane organelles, mitochondria have an outer mitochondrial membrane (OMM), where proteins responsible for transport of different molecules are embedded [76]; an intermembrane space (IMS) and an inner mitochondrial membrane (IMM), where electron transport chain (ETC) proteins are localized and oxidative phosphorylation (OXPHOS) and ATP production takes place [77], and a mitochondrial matrix (MM), compartment, where many metabolic pathways occur, such as the tricarboxylic acid cycle, fatty-acid oxidation, synthesis of biomolecules and regulation of apoptosis [78]. The proper development of mitochondrial processes is critical for immune response, as the susceptibility to microbial infections and the risk of systemic inflammatory responses increases considerably when these organelles malfunction [79,80].

Mitochondria are important for antiviral signaling. During RNA-virus infection, viral RNAs are initially recognized by cytoplasmic sensors, mainly RIG-I-like receptors (RLRs) [2], whose interaction with mitochondria is essential for the coordination and development of an adequate antiviral response. The common structure of RLRs consists of a carboxy-terminal regulatory domain, a central RNA helicase domain and amino-terminal caspase recruitment domains (CARDs) [81]. After binding to viral RNA, RLRs trigger IFN-mediated antiviral responses through their interaction with mitochondrial antiviral-signaling protein (MAVS), a CARD-containing OMM protein [82]. The CARD-CARD interaction between RLRs and MAVS causes MAVS polymerization and consequent recruitment of a variety of downstream effectors, including tumor necrosis factor receptor-associated factor family proteins, IKB kinase *epsilon* (IKKε) and TANK binding kinase 1, among others [83]. This “MAVS signalosome” activates NF-κB, IRF3 and IRF7, promoting the expression of type-I IFN and antiviral molecules [84]. Given the central role of MAVS in mitochondrial antiviral signaling, MAVS and both upstream and downstream molecules are under tight regulation to ensure an adequate response [85,86].

Mitochondria are dynamic organelles that undergo constant fusion and fission to regulate their morphology, activity and turnover according to the metabolic needs of the cell [87], and these mitochondrial dynamics are involved in the regulation of mitochondrial immune functions. Mitofusins and optic atrophy protein 1 are responsible for mitochondrial fusion, whereas the cytosolic GTP-ase dynamin-related protein 1 (Drp1) mediates mitochondrial fission through its interaction with adaptor proteins in the OMM [88,89]. Interestingly, these proteins have been shown to be implicated in the regulation of various mitochondrial immune-relevant processes, such as RLR signaling [83,90,91], apoptosis [92,93], autophagy and mitochondrial bioenergetic conditions [94], which are important mechanisms to combat viral infections.

Mitophagy is a selective autophagic process in which defective mitochondria are engulfed in autophagosomes and eliminated by fusion with lysosomes [95]. Damaged mitochondria constitute a signal for the recruitment of PTEN-induced putative kinase protein 1 (PINK1), which surrounds the mitochondrial surface. The accumulation of PINK1 and its kinase activity promote the translocation of the E3 ubiquitin ligase Parkin from the cytosol to dysfunctional mitochondria, triggering the ubiquitylation of OMM proteins. The formation of ubiquitin chains by Parkin favors the binding of adaptor proteins (e.g., p62 and optineurin), which mediate the interaction with autophagosomes and the further degradation of dysfunctional mitochondria [96,97]. Mitophagy is closely related to mitochondrial dynamics, as mitochondrial fragmentation promotes mitophagy whereas mitochondrial fusion hinders this process [98,99]. Interestingly, defective mitochondria can play either positive or negative roles against viruses. For example, alterations in mitochondrial respiration trigger the production of reactive oxygen species (ROS) which, in addition to being harmful for the cell in high levels, play an important role as second messengers in diverse intracellular signaling pathways [100]. In the context of antiviral signaling, ROS are involved in the regulation of the RLR pathway, potentiating RLR-MAVS signaling and the production of type-I IFN [101]. Because healthy mitochondria are required for an adequate metabolic state and the activation of apoptotic processes [94,102,103], mitophagy must be finely regulated to modulate the mitochondria-mediated antiviral response.

The implications of mitochondria in innate immunity are enormous. We have briefly discussed the interplay between different mitochondrial pathways, such as RLR signaling, mitochondrial dynamics, mitophagy, ROS production and apoptosis, in the protection against viruses. However, mitochondria perform a plethora of functions in the establishment of a defensive state of the cell, which have been thoroughly reviewed by others [85,104,105,106,107,108]. Moreover, the regulation of mitochondrial function is not only carried out by the host, but also by viruses with the aim to shut down defense mechanisms, complete their life cycle and spread [109], underscoring the relevance of mitochondria in antiviral response. Such critical roles of mitochondria in the control of pathogen invasion and maintenance of cellular homeostasis must be strictly coordinated.

### 2.1. Mitochondria: Targets of Ubiquitin-Like Modifications

As we previously discussed, ubiquitin and ubiquitin-like PTMs are key regulatory processes of the innate and adaptive immune response against viruses, and both processes are finely regulated by mitochondria. In this regard, there is a broad spectrum of PTMs [110], some of which occur within mitochondria, which are responsible for modifying their internal state and function [111,112]. Thus, mitochondria are targets of ubiquitin and ubiquitin-like proteins, such as small ubiquitin-like modifiers (SUMOs) and ISG15. 

Ubiquitin is a highly conserved 8.6-kDa protein known as a master regulator of cellular processes. Its covalent conjugation to target proteins has proteolytic and non-proteolytic or regulatory outcomes, which fine-tune protein function and recycling [113]. Indeed, ubiquitylation is essential for the regulation of many mitochondrial processes, such as mitophagy [95,114,115,116], mitochondrial dynamics [117,118], and mitochondria-related immune signaling [86,119,120], establishing the importance of this modifier in the homeostasis of these organelles.

SUMOs are a family of highly conserved 12-kDa proteins that are essential in eukaryotic cells. Similar to ubiquitin, SUMO conjugation to specific lysine residues of target proteins (SUMOylation) alters their function, their interaction with other proteins, and their stability [121,122]. Although the major role of SUMOylation is in the regulation of nuclear processes [123], it also targets mitochondria. SUMOs have been proven to be involved in the regulation of mitochondrial dynamics by binding to Drp1 [124], with an important implication in programmed cell death [125,126,127]. Furthermore, SUMOylation of the mitochondrial oxidative stress sensor DJ-1 results in its stabilization and full activation, reinforcing its protective role against Parkinson’s disease [128], where mitochondrial dysfunction has great significance.

#### ISG15 and Mitochondria

Although ISG15 has been associated with mitochondria, its functions, both free or conjugated to mitochondrial proteins, are still being examined. One exception is the protein Parkin. While not strictly a mitochondrial protein, its translocation to the OMM from the cytoplasm is essential for Parkin-mediated mitophagy [96]. ISG15 conjugation to Parkin enhances its E3 ubiquitin ligase activity and its cytoprotective effect in Parkinson’s disease [129], an example of how ISGylation affects mitochondrial processes.

**Figure 3:**
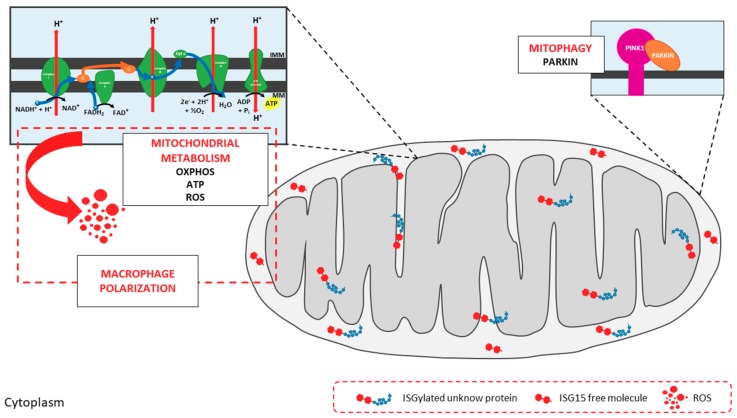
*Impact of ISG15 on mitochondrial activities.* Mitochondria are targets of ISG15 and ISGylation in murine bone marrow-derived macrophages (BMDMs). ISGylated proteins can be found in all mitochondrial localizations, mainly in the mitochondrial intermembrane space (IMS) and inner mitochondrial membrane (IMM), where free ISG15 is also present. ISG15 and ISGylation are involved in the regulation of mitochondrial metabolism. Absence of ISG15 leads to alterations in OXPHOS, with lower oxygen consumption rates and ATP production levels, in addition to aberrant ETC supercomplexes assembly. Such disruption of OXPHOS mechanisms decreases ROS production, with repercussions for macrophage polarization. Mitophagy is also altered in cells lacking ISG15. Finally, *ISG15*-/- BMDM accumulate defective mitochondria and Parkin cannot be found in mitochondrial extracts, suggesting that ISG15 is important during the translocation of Parkin from the cytoplasm to mitochondria.

ISG15 and ISGylation for the regulation of mitochondrial metabolism [29]. We undertook a comprehensive analysis of bone marrow-derived macrophages (BMDMs) from wild-type and *ISG15*-/- mice to interrogate how ISG15 and ISGylation could modulate the regulation of mitochondria in the context of stressful stimuli. Monomeric ISG15 and ISGylated proteins were observed in mitochondrial fractions from wild-type BMDMs after type-I IFN pre-treatment, and these proteins were preferentially located to the IMS and IMM (Figure 3). Given their localization, we hypothesized that ISG15 and ISGylation could impact mitochondrial respiratory metabolism, and we focused our study on OXPHOS and ATP production. This analysis revealed that oxygen consumption and ATP production were lower in *ISG15*-/- BMDMs than in equivalent wild-type cells, indicative of defective OXPHOS. In accord with these observations, a clear difference in the distribution of ETC supercomplexes was observed between the two groups, pointing to a possible role for ISG15 in the correct assembly of ETC proteins (Figure 3). As recently reported by Yoshizumi et al. [130] OXPHOS activity is required for RLR-mediated antiviral signaling, and mice with OXPHOS defects showed increased susceptibility to viral infections. Similarly, knockout mice for ISG15 or the ISG15-activating E1 enzyme (Ube1L) were more susceptible to infection with many viruses than wild-type mice [30]. Since the lack of ISG15 seems to cause alterations in OXPHOS, such increase in the sensitivity to viral infections in *ISG15*-/- and Ube1L-/- mice might be explained as a result of defects in RLR-mediated antiviral responses, supporting the role of ISG15 as a regulator of mitochondrial functions. Regarding mitochondrial respiration byproducts, *ISG15*-/- BMDM also produced lower levels of ROS. Because ROS production is tightly controlled by mitochondrial membrane potential [131], low levels of ROS in *ISG15*-/- BMDM might be the result of abnormalities in the transmembrane proton gradient due to the absence of ISG15, and could affect the immune response against viral infections, as discussed earlier. Mitochondrial ROS also participate in the regulation of macrophage polarization [132] and, interestingly, *ISG15*-/- BMDMs displayed mixed features of M1 and M2 phenotypes, suggesting that alterations in mitochondrial OXPHOS could drive changes to immune cell function. Finally, BMDMs lacking ISG15 accumulated non-functional mitochondria with an absence of Parkin, suggesting that ISG15 is also implicated in the regulation of mitophagy, perhaps through the control of Parkin translocation from the cytosol (Figure 3).

Taken together, these findings establish a relevant role for ISG15 and ISGylation in the control of mitochondrial OXPHOS and recycling, at least in murine BMDM, expanding the range of functions of this PTM and underscoring its importance in the regulation of essential cellular processes. 

## 3. Future Perspectives

The functional significance of PTMs in disease etiology, and the pathologic response to their disruption, is the subject of intense investigation. Many of these reversible modifications act as regulatory mechanisms in mitochondria and show promise for mitochondria-targeted therapeutic strategies. With the advent of mass spectrometry-based screening techniques, there has been a vast increase in our current state of knowledge on mitochondrial PTMs and their protein targets. Detecting ISGylated proteins in different organelles remains challenging, as it typically occurs in only a small portion of the total protein pool of the cell, albeit with essential roles in regulating protein fate and function. Understanding the consequences of ISGylation of mitochondrial proteins will require much work, but should be rewarding not only for developing new strategies to combat viral infections, but also for future applications in other biomedically relevant processes/diseases, for example inflammation, cancer and neurodegeneration.

## Figures and Tables

**Table 1:** ISGylated proteins predicted to locate to mitochondria. Proteins identified as ISGylation targets in different proteomic studies [60,68,69,70] predicted to locate to mitochondria. Proteins are grouped according to biological functions.

Biological Function	Potentially ISGylated Mitocondrial Proteins
Host-virus interaction	Acyl-CoA thioesterase 8 (ACOT8) [60]
	Complement C1q binding protein (C1QBP) [69]
	Receptor for activated C kinase 1 (RACK1) [60]
	Solute carrier family 25 member 5 (SLC25A5) [69]
	Solute carrier family 25 member 6 (SLC25A6) [69]
	Staphylococcal nuclease and tudor domain containing 1 (SND1) [69]
Negative regulation of apoptotic process	NME/NM23 nucleoside diphosphate kinase 2 (NME2) [69]
	Annexin A1 (ANXA1) [69,70]
	Glutathione S-transferase pi 1 (GSTP1) [60]
	Heat shock protein family A (Hsp70) member 5 (HSPA5) [69]
	Interferon-induced protein with tetratricopeptide repeats 3 (IFIT3) [60]
Positive regulation of protein insertion into mitochondrial membrane involved in apoptotic signaling pathway	Stratifin (SFN) [69]
	Tyrosine 3-monooxygenase/tryptophan 5-monooxygenase activation protein beta (YWHAB) [69]
	Tyrosine 3-monooxygenase/tryptophan 5-monooxygenase activation protein épsilon (YWHAE) [69]
	Tyrosine 3-monooxygenase/tryptophan 5-monooxygenase activation protein gamma (YWHAG) [69]
	Tyrosine 3-monooxygenase/tryptophan 5-monooxygenase activation protein theta (YWHAQ) [69]
	Tyrosine 3-monooxygenase/tryptophan 5-monooxygenase activation protein zeta (YWHAZ) [69]
ATP biosynthetic process	ATP synthase, H+ transporting, mitochondrial F1 complex, alpha subunit 1, cardiac muscle (ATP5A1) [60,69]
	ATP synthase, H+ transporting, mitochondrial F1 complex, beta polypeptide (ATP5B) [60,69]
	ATP synthase, H+ transporting, mitochondrial Fo complex subunit G (ATP5L) [70]
Oxidation-reduction process	Aldehyde dehydrogenase 18 family member A1 (ALDH18A1) [70]
	Fatty acid synthase (FASN) [60,69]
	Glutathione-disulfide reductase (GSR) [69]
	Lactate dehydrogenase B (LDHB) [69]
	Malic enzyme 1 (ME1) [68]
	Peroxiredoxin 1 (PRDX1) [60,69,70]
	Peroxiredoxin 4 (PRDX4) [69]
	Sorbitol dehydrogenase (SORD) [68]
	Superoxide dismutase 1, soluble(SOD1) [69]
	Thioredoxin reductase 1 (TXNRD1) [60,69]
	Thioredoxin (TXN) [69]
Aminoacyl-tRNA synthetase	Alanyl-tRNA synthetase (AARS) [68]
	Glycyl-tRNA synthetase (GARS) [68]
	Phenylalanyl-tRNA synthetase 2, mitocondrial (FARS2) [60]
Tricarboxylic acid cycle	Malate dehydrogenase 1 (MDH1) [69]
	Malate dehydrogenase 2 (MDH2) [69]
Glycolisis	Oxoglutarate dehydrogenase (OGDH) [60]
	Pyruvate kinase, muscle (PKM) [60,69,70]
Chaperone	Chaperonin containing TCP1 subunit 7 (CCT7) [69]
	Heat shock protein 90 alpha family class B member 1 (HSP90AB1) [60,69,70]
	Heat shock protein family A (Hsp70) member 1A (HSPA1A) [60,69]
	Heat shock protein family D (Hsp60) member 1 (HSPD1) [60,69]
Ion channel	Chloride intracellular channel 1 (CLIC1) [60,69]
	Annexin A6 (ANXA6) [69]
Other functions	Creatine kinase, mitochondrial 1B (CKMT1B) [69]
	Ubiquitin-like modifier activating enzyme 1 (UBA1) [69]
	Leucine aminopeptidase 3 (LAP3) [60]
	5-aminoimidazole-4-carboxamide ribonucleotide formyltransferase/IMP cyclohydrolase (ATIC) [60,69]
	clathrin heavy chain (CLTC) [60,69]
	Queuine tRNA-ribosyltransferase accessory subunit 2 (QTRT2) [60]
	Enoyl-CoA hydratase and 3-hydroxyacyl CoA dehydrogenase (EHHADH) [69]
	ATP binding cassette subfamily F member 2 (ABCF2) [60]

## References

[1] Iwasaki A. (2012). A virological view of innate immune recognition. Annu. Rev. Microbiol..

[2] Brubaker S.W., Bonham K.S., Zanoni I., Kagan J.C. (2015). Innate immune pattern recognition: A cell biological perspective. Annu. Rev. Immunol..

[3] Ivashkiv L.B., Donlin L.T. (2014). Regulation of type I interferon responses. Nat. Rev. Immunol..

[4] Raftery N., Stevenson N.J. (2017). Advances in anti-viral immune defence: Revealing the importance of the IFN JAK/STAT pathway. Cell. Mol. Life Sci..

[5] Zhang D., Zhang D.E. (2011). Interferon-stimulated gene 15 and the protein ISGylation system. J. Interferon Cytokine Res..

[6] Potter J.L., Narasimhan J., Mende-Mueller L., Haas A.L. (1999). Precursor processing of pro-ISG15/UCRP, an interferon-beta-induced ubiquitin-like protein. J. Biol. Chem..

[7] Durfee L.A., Huibregtse J.M. (2012). The ISG15 conjugation system. Methods Mol. Biol..

[8] Durfee L.A., Lyon N., Seo K., Huibregtse J.M. (2010). The ISG15 conjugation system broadly targets newly synthesized proteins: Implications for the antiviral function of ISG15. Mol. Cell.

[9] Malakhov M.P., Kim K.I., Malakhova O.A., Jacobs B.S., Borden E.C., Zhang D.E. (2003). High-throughput immunoblotting. Ubiquitiin-like protein ISG15 modifies key regulators of signal transduction. J. Biol. Chem..

[10] Villarroya-Beltri C., Guerra S., Sanchez-Madrid F. (2017). ISGylation—A key to lock the cell gates for preventing the spread of threats. J. Cell Sci..

[11] Taylor J.L., D’Cunha J., Tom P., O’Brien W.J., Borden E.C. (1996). Production of ISG-15, an interferon-inducible protein, in human corneal cells. J. Interferon Cytokine Res..

[12] Cai B., Bai Q., Chi X., Goraya M.U., Wang L., Wang S., Chen B., Chen J.L. (2017). Infection with Classical Swine Fever Virus Induces Expression of Type III Interferons and Activates Innate Immune Signaling. Front. Microbiol..

[13] Tecalco Cruz A.C., Mejia-Barreto K. (2017). Cell type-dependent regulation of free ISG15 levels and ISGylation. J. Cell Commun. Signal..

[14] Malakhova O., Malakhov M., Hetherington C., Zhang D.E. (2002). Lipopolysaccharide activates the expression of ISG15-specific protease UBP43 via interferon regulatory factor 3. J. Biol. Chem..

[15] Pitha-Rowe I., Hassel B.A., Dmitrovsky E. (2004). Involvement of UBE1L in ISG15 conjugation during retinoid-induced differentiation of acute promyelocytic leukemia. J. Biol. Chem..

[16] Jeon Y.J., Park J.H., Chung C.H. (2017). Interferon-Stimulated Gene 15 in the Control of Cellular Responses to Genotoxic Stress. Mol. Cells.

[17] Honke N., Shaabani N., Zhang D.E., Hardt C., Lang K.S. (2016). Multiple functions of USP18. Cell Death Dis..

[18] Zhang X., Bogunovic D., Payelle-Brogard B., Francois-Newton V., Speer S.D., Yuan C., Volpi S., Li Z., Sanal O., Mansouri D. (2015). Human intracellular ISG15 prevents interferon-alpha/beta over-amplification and auto-inflammation. Nature.

[19] Speer S.D., Li Z., Buta S., Payelle-Brogard B., Qian L., Vigant F., Rubino E., Gardner T.J., Wedeking T., Hermann M. (2016). ISG15 deficiency and increased viral resistance in humans but not mice. Nat. Commun..

[20] Bogunovic D., Byun M., Durfee L.A., Abhyankar A., Sanal O., Mansouri D., Salem S., Radovanovic I., Grant A.V., Adimi P. (2012). Mycobacterial disease and impaired IFN-gamma immunity in humans with inherited ISG15 deficiency. Science.

[21] Swaim C.D., Scott A.F., Canadeo L.A., Huibregtse J.M. (2017). Extracellular ISG15 Signals Cytokine Secretion through the LFA-1 Integrin Receptor. Mol. Cell.

[22] D’Cunha J., Knight E., Haas A.L., Truitt R.L., Borden E.C. (1996). Immunoregulatory properties of ISG15, an interferon-induced cytokine. Proc. Natl. Acad. Sci. USA.

[23] Padovan E., Terracciano L., Certa U., Jacobs B., Reschner A., Bolli M., Spagnoli G.C., Borden E.C., Heberer M. (2002). Interferon stimulated gene 15 constitutively produced by melanoma cells induces e-cadherin expression on human dendritic cells. Cancer Res..

[24] Owhashi M., Taoka Y., Ishii K., Nakazawa S., Uemura H., Kambara H. (2003). Identification of a ubiquitin family protein as a novel neutrophil chemotactic factor. Biochem. Biophys. Res. Commun..

[25] Napolitano A., van der Veen A.G., Bunyan M., Borg A., Frith D., Howell S., Kjaer S., Beling A., Snijders A.P., Knobeloch K.P. (2018). Cysteine-Reactive Free ISG15 Generates IL-1beta-Producing CD8alpha(+) Dendritic Cells at the Site of Infection. J. Immunol..

[26] Takeuchi T., Iwahara S., Saeki Y., Sasajima H., Yokosawa H. (2005). Link between the ubiquitin conjugation system and the ISG15 conjugation system: ISG15 conjugation to the UbcH6 ubiquitin E2 enzyme. J. Biochem..

[27] Okumura A., Pitha P.M., Harty R.N. (2008). ISG15 inhibits Ebola VP40 VLP budding in an L-domain-dependent manner by blocking Nedd4 ligase activity. Proc. Natl. Acad. Sci. USA.

[28] Fan J.B., Arimoto K., Motamedchaboki K., Yan M., Wolf D.A., Zhang D.E. (2015). Identification and characterization of a novel ISG15-ubiquitin mixed chain and its role in regulating protein homeostasis. Sci. Rep..

[29] Baldanta S., Fernandez-Escobar M., Acin-Perez R., Albert M., Camafeita E., Jorge I., Vazquez J., Enriquez J.A., Guerra S. (2017). ISG15 governs mitochondrial function in macrophages following vaccinia virus infection. PLoS Pathog..

[30] Perng Y.C., Lenschow D.J. (2018). ISG15 in antiviral immunity and beyond. Nat. Rev. Microbiol..

[31] Lenschow D.J., Lai C., Frias-Staheli N., Giannakopoulos N.V., Lutz A., Wolff T., Osiak A., Levine B., Schmidt R.E., Garcia-Sastre A. (2007). IFN-stimulated gene 15 functions as a critical antiviral molecule against influenza, herpes, and Sindbis viruses. Proc. Natl. Acad. Sci. USA.

[32] Giannakopoulos N.V., Arutyunova E., Lai C., Lenschow D.J., Haas A.L., Virgin H.W. (2009). ISG15 Arg151 and the ISG15-conjugating enzyme UbE1L are important for innate immune control of Sindbis virus. J. Virol..

[33] Lenschow D.J., Giannakopoulos N.V., Gunn L.J., Johnston C., O’Guin A.K., Schmidt R.E., Levine B., Virgin H.W.T. (2005). Identification of interferon-stimulated gene 15 as an antiviral molecule during Sindbis virus infection in vivo. J. Virol..

[34] Werneke S.W., Schilte C., Rohatgi A., Monte K.J., Michault A., Arenzana-Seisdedos F., Vanlandingham D.L., Higgs S., Fontanet A., Albert M.L. (2011). ISG15 is critical in the control of Chikungunya virus infection independent of UbE1L mediated conjugation. PLoS Pathog..

[35] Kim J.H., Luo J.K., Zhang D.E. (2008). The level of hepatitis B virus replication is not affected by protein ISG15 modification but is reduced by inhibition of UBP43 (USP18) expression. J. Immunol..

[36] Ritchie K.J., Hahn C.S., Kim K.I., Yan M., Rosario D., Li L., de la Torre J.C., Zhang D.E. (2004). Role of ISG15 protease UBP43 (USP18) in innate immunity to viral infection. Nat. Med..

[37] Knobeloch K.P., Utermohlen O., Kisser A., Prinz M., Horak I. (2005). Reexamination of the role of ubiquitin-like modifier ISG15 in the phenotype of UBP43-deficient mice. Mol. Cell. Biol..

[38] Moore E.C., Barber J., Tripp R.A. (2008). Respiratory syncytial virus (RSV) attachment and nonstructural proteins modify the type I interferon response associated with suppressor of cytokine signaling (SOCS) proteins and IFN-stimulated gene-15 (ISG15). Virol. J..

[39] Gonzalez-Sanz R., Mata M., Bermejo-Martin J., Alvarez A., Cortijo J., Melero J.A., Martinez I. (2016). ISG15 Is Upregulated in Respiratory Syncytial Virus Infection and Reduces Virus Growth through Protein ISGylation. J. Virol..

[40] Okumura A., Lu G., Pitha-Rowe I., Pitha P.M. (2006). Innate antiviral response targets HIV-1 release by the induction of ubiquitin-like protein ISG15. Proc. Natl. Acad. Sci. USA.

[41] Langevin C., van der Aa L.M., Houel A., Torhy C., Briolat V., Lunazzi A., Harmache A., Bremont M., Levraud J.P., Boudinot P. (2013). Zebrafish ISG15 exerts a strong antiviral activity against RNA and DNA viruses and regulates the interferon response. J. Virol..

[42] Yuan W., Krug R.M. (2001). Influenza B virus NS1 protein inhibits conjugation of the interferon (IFN)-induced ubiquitin-like ISG15 protein. EMBO J..

[43] Guerra S., Caceres A., Knobeloch K.P., Horak I., Esteban M. (2008). Vaccinia virus E3 protein prevents the antiviral action of ISG15. PLoS Pathog..

[44] Kim Y.J., Kim E.T., Kim Y.E., Lee M.K., Kwon K.M., Kim K.I., Stamminger T., Ahn J.H. (2016). Consecutive Inhibition of ISG15 Expression and ISGylation by Cytomegalovirus Regulators. PLoS Pathog..

[45] Frias-Staheli N., Giannakopoulos N.V., Kikkert M., Taylor S.L., Bridgen A., Paragas J., Richt J.A., Rowland R.R., Schmaljohn C.S., Lenschow D.J. (2007). Ovarian tumor domain-containing viral proteases evade ubiquitin- and ISG15-dependent innate immune responses. Cell Host Microbe.

[46] Deng X., Agnihothram S., Mielech A.M., Nichols D.B., Wilson M.W., StJohn S.E., Larsen S.D., Mesecar A.D., Lenschow D.J., Baric R.S. (2014). A chimeric virus-mouse model system for evaluating the function and inhibition of papain-like proteases of emerging coronaviruses. J. Virol..

[47] Arnaud N., Dabo S., Akazawa D., Fukasawa M., Shinkai-Ouchi F., Hugon J., Wakita T., Meurs E.F. (2011). Hepatitis C virus reveals a novel early control in acute immune response. PLoS Pathog..

[48] Chen L., Li S., McGilvray I. (2011). The ISG15/USP18 ubiquitin-like pathway (ISGylation system) in hepatitis C virus infection and resistance to interferon therapy. Int. J. Biochem. Cell Biol..

[49] Marc D. (2014). Influenza virus non-structural protein NS1: Interferon antagonism and beyond. J. Gen. Virol..

[50] Tang Y., Zhong G., Zhu L., Liu X., Shan Y., Feng H., Bu Z., Chen H., Wang C. (2010). Herc5 attenuates influenza A virus by catalyzing ISGylation of viral NS1 protein. J. Immunol..

[51] Zhao C., Hsiang T.Y., Kuo R.L., Krug R.M. (2010). ISG15 conjugation system targets the viral NS1 protein in influenza A virus-infected cells. Proc. Natl. Acad. Sci. USA.

[52] Zhao C., Sridharan H., Chen R., Baker D.P., Wang S., Krug R.M. (2016). Influenza B virus non-structural protein 1 counteracts ISG15 antiviral activity by sequestering ISGylated viral proteins. Nat. Commun..

[53] Rahnefeld A., Klingel K., Schuermann A., Diny N.L., Althof N., Lindner A., Bleienheuft P., Savvatis K., Respondek D., Opitz E. (2014). Ubiquitin-like protein ISG15 (interferon-stimulated gene of 15 kDa) in host defense against heart failure in a mouse model of virus-induced cardiomyopathy. Circulation.

[54] Nakashima H., Nguyen T., Goins W.F., Chiocca E.A. (2015). Interferon-stimulated gene 15 (ISG15) and ISG15-linked proteins can associate with members of the selective autophagic process, histone deacetylase 6 (HDAC6) and SQSTM1/p62. J. Biol. Chem..

[55] Desai S.D., Haas A.L., Wood L.M., Tsai Y.C., Pestka S., Rubin E.H., Saleem A., Nur E.K.A., Liu L.F. (2006). Elevated expression of ISG15 in tumor cells interferes with the ubiquitin/26S proteasome pathway. Cancer Res..

[56] Liu M., Li X.L., Hassel B.A. (2003). Proteasomes modulate conjugation to the ubiquitin-like protein, ISG15. J. Biol. Chem..

[57] Ganesan M., Poluektova L.Y., Tuma D.J., Kharbanda K.K., Osna N.A. (2016). Acetaldehyde Disrupts Interferon Alpha Signaling in Hepatitis C Virus-Infected Liver Cells by Up-Regulating USP18. Alcohol. Clin. Exp. Res..

[58] Okumura F., Okumura A.J., Uematsu K., Hatakeyama S., Zhang D.E., Kamura T. (2013). Activation of double-stranded RNA-activated protein kinase (PKR) by interferon-stimulated gene 15 (ISG15) modification down-regulates protein translation. J. Biol. Chem..

[59] Kim M.J., Hwang S.Y., Imaizumi T., Yoo J.Y. (2008). Negative feedback regulation of RIG-I-mediated antiviral signaling by interferon-induced ISG15 conjugation. J. Virol..

[60] Zhao C., Denison C., Huibregtse J.M., Gygi S., Krug R.M. (2005). Human ISG15 conjugation targets both IFN-induced and constitutively expressed proteins functioning in diverse cellular pathways. Proc. Natl. Acad. Sci. USA.

[61] Du Y., Duan T., Feng Y., Liu Q., Lin M., Cui J., Wang R.F. (2018). LRRC25 inhibits type I IFN signaling by targeting ISG15-associated RIG-I for autophagic degradation. EMBO J..

[62] Malakhova O.A., Yan M., Malakhov M.P., Yuan Y., Ritchie K.J., Kim K.I., Peterson L.F., Shuai K., Zhang D.E. (2003). Protein ISGylation modulates the JAK-STAT signaling pathway. Genes Dev..

[63] Shi H.X., Yang K., Liu X., Liu X.Y., Wei B., Shan Y.F., Zhu L.H., Wang C. (2010). Positive regulation of interferon regulatory factor 3 activation by Herc5 via ISG15 modification. Mol. Cell. Biol..

[64] Jeon Y.J., Choi J.S., Lee J.Y., Yu K.R., Kim S.M., Ka S.H., Oh K.H., Kim K.I., Zhang D.E., Bang O.S. (2009). ISG15 modification of filamin B negatively regulates the type I interferon-induced JNK signalling pathway. EMBO Rep..

[65] Pincetic A., Kuang Z., Seo E.J., Leis J. (2010). The interferon-induced gene ISG15 blocks retrovirus release from cells late in the budding process. J. Virol..

[66] Sanyal S., Ashour J., Maruyama T., Altenburg A.F., Cragnolini J.J., Bilate A., Avalos A.M., Kundrat L., Garcia-Sastre A., Ploegh H.L. (2013). Type I interferon imposes a TSG101/ISG15 checkpoint at the Golgi for glycoprotein trafficking during influenza virus infection. Cell Host Microbe.

[67] Villarroya-Beltri C., Baixauli F., Mittelbrunn M., Fernandez-Delgado I., Torralba D., Moreno-Gonzalo O., Baldanta S., Enrich C., Guerra S., Sanchez-Madrid F. (2016). ISGylation controls exosome secretion by promoting lysosomal degradation of MVB proteins. Nat. Commun..

[68] Giannakopoulos N.V., Luo J.K., Papov V., Zou W., Lenschow D.J., Jacobs B.S., Borden E.C., Li J., Virgin H.W., Zhang D.E. (2005). Proteomic identification of proteins conjugated to ISG15 in mouse and human cells. Biochem. Biophys. Res. Commun..

[69] Wong J.J., Pung Y.F., Sze N.S., Chin K.C. (2006). HERC5 is an IFN-induced HECT-type E3 protein ligase that mediates type I IFN-induced ISGylation of protein targets. Proc. Natl. Acad. Sci. USA.

[70] Peng Q.-S., Li G.-P., Sun W.-C., Yang J.-B., Quan G.-H., Liu N. (2016). Analysis of ISG15-Modified Proteins from A549 Cells in Response to Influenza Virus Infection by Liquid Chromatography-Tandem Mass Spectrometry. Chin. J. Anal. Chem..

[71] Huang D.W., Sherman B.T., Lempicki R.A. (2009). Systematic and integrative analysis of large gene lists using DAVID bioinformatics resources. Nat. Protoc..

[72] Huang D.W., Sherman B.T., Lempicki R.A. (2009). Bioinformatics enrichment tools: Paths toward the comprehensive functional analysis of large gene lists. Nucleic Acids Res..

[73] Austin K.J., Carr A.L., Pru J.K., Hearne C.E., George E.L., Belden E.L., Hansen T.R. (2004). Localization of ISG15 and conjugated proteins in bovine endometrium using immunohistochemistry and electron microscopy. Endocrinology.

[74] Shadel G.S., Clayton D.A. (1997). Mitochondrial DNA maintenance in vertebrates. Annu. Rev. Biochem..

[75] Neupert W., Herrmann J.M. (2007). Translocation of proteins into mitochondria. Annu. Rev. Biochem..

[76] Becker T., Wagner R. (2018). Mitochondrial Outer Membrane Channels: Emerging Diversity in Transport Processes. Bioessays.

[77] Papa S., Martino P.L., Capitanio G., Gaballo A., De Rasmo D., Signorile A., Petruzzella V. (2012). The oxidative phosphorylation system in mammalian mitochondria. Adv. Exp. Med. Biol..

[78] Cannino G., Ciscato F., Masgras I., Sanchez-Martin C., Rasola A. (2018). Metabolic Plasticity of Tumor Cell Mitochondria. Front. Oncol..

[79] Singer M. (2014). The role of mitochondrial dysfunction in sepsis-induced multi-organ failure. Virulence.

[80] Shinde A., Luo J., Bharathi S.S., Shi H., Beck M.E., McHugh K.J., Alcorn J.F., Wang J., Goetzman E.S. (2018). Increased mortality from influenza infection in long-chain acyl-CoA dehydrogenase knockout mice. Biochem. Biophys. Res. Commun..

[81] Kawai T., Akira S. (2009). The roles of TLRs, RLRs and NLRs in pathogen recognition. Int. Immunol..

[82] Seth R.B., Sun L., Ea C.K., Chen Z.J. (2005). Identification and characterization of MAVS, a mitochondrial antiviral signaling protein that activates NF-kappaB and IRF 3. Cell.

[83] Koshiba T., Yasukawa K., Yanagi Y., Kawabata S. (2011). Mitochondrial membrane potential is required for MAVS-mediated antiviral signaling. Sci. Signal..

[84] Hou F., Sun L., Zheng H., Skaug B., Jiang Q.X., Chen Z.J. (2011). MAVS forms functional prion-like aggregates to activate and propagate antiviral innate immune response. Cell.

[85] Jin H.S., Suh H.W., Kim S.J., Jo E.K. (2017). Mitochondrial Control of Innate Immunity and Inflammation. Immune Netw..

[86] Liu B., Gao C. (2018). Regulation of MAVS activation through post-translational modifications. Curr. Opin. Immunol..

[87] Friedman J.R., Nunnari J. (2014). Mitochondrial form and function. Nature.

[88] Lee H., Yoon Y. (2014). Mitochondrial fission: Regulation and ER connection. Mol. Cells.

[89] Tilokani L., Nagashima S., Paupe V., Prudent J. (2018). Mitochondrial dynamics: Overview of molecular mechanisms. Essays Biochem..

[90] Castanier C., Garcin D., Vazquez A., Arnoult D. (2010). Mitochondrial dynamics regulate the RIG-I-like receptor antiviral pathway. EMBO Rep..

[91] Onoguchi K., Onomoto K., Takamatsu S., Jogi M., Takemura A., Morimoto S., Julkunen I., Namiki H., Yoneyama M., Fujita T. (2010). Virus-infection or 5’ppp-RNA activates antiviral signal through redistribution of IPS-1 mediated by MFN1. PLoS Pathog..

[92] Arnoult D. (2007). Mitochondrial fragmentation in apoptosis. Trends Cell Biol..

[93] Castanier C., Arnoult D. (2010). Mitochondrial dynamics during apoptosis. Med. Sci..

[94] Schrepfer E., Scorrano L. (2016). Mitofusins, from Mitochondria to Metabolism. Mol. Cell.

[95] Pickles S., Vigie P., Youle R.J. (2018). Mitophagy and Quality Control Mechanisms in Mitochondrial Maintenance. Curr. Biol..

[96] Youle R.J., Narendra D.P. (2011). Mechanisms of mitophagy. Nat. Rev. Mol. Cell Biol..

[97] Gkikas I., Palikaras K., Tavernarakis N. (2018). The Role of Mitophagy in Innate Immunity. Front. Immunol..

[98] Chan D.C. (2006). Mitochondria: Dynamic organelles in disease, aging, and development. Cell.

[99] Twig G., Shirihai O.S. (2011). The interplay between mitochondrial dynamics and mitophagy. Antioxid. Redox Signal..

[100] Hamanaka R.B., Chandel N.S. (2010). Mitochondrial reactive oxygen species regulate cellular signaling and dictate biological outcomes. Trends Biochem. Sci..

[101] Tal M.C., Sasai M., Lee H.K., Yordy B., Shadel G.S., Iwasaki A. (2009). Absence of autophagy results in reactive oxygen species-dependent amplification of RLR signaling. Proc. Natl. Acad. Sci. USA.

[102] Elmore S. (2007). Apoptosis: A review of programmed cell death. Toxicol. Pathol..

[103] Osellame L.D., Blacker T.S., Duchen M.R. (2012). Cellular and molecular mechanisms of mitochondrial function. Best Pract. Res. Clin. Endocrinol. Metab..

[104] Weinberg S.E., Sena L.A., Chandel N.S. (2015). Mitochondria in the regulation of innate and adaptive immunity. Immunity.

[105] Mills E.L., Kelly B., O’Neill L.A.J. (2017). Mitochondria are the powerhouses of immunity. Nat. Immunol..

[106] Sandhir R., Halder A., Sunkaria A. (2017). Mitochondria as a centrally positioned hub in the innate immune response. Biochim. Biophys. Acta Mol. Basis Dis..

[107] Angajala A., Lim S., Phillips J.B., Kim J.H., Yates C., You Z., Tan M. (2018). Diverse Roles of Mitochondria in Immune Responses: Novel Insights Into Immuno-Metabolism. Front. Immunol..

[108] Banoth B., Cassel S.L. (2018). Mitochondria in innate immune signaling. Transl. Res..

[109] Anand S.K., Tikoo S.K. (2013). Viruses as modulators of mitochondrial functions. Adv. Virol..

[110] Vu L.D., Gevaert K., De Smet I. (2018). Protein Language: Post-Translational Modifications Talking to Each Other. Trends Plant Sci..

[111] Marquez J., Lee S.R., Kim N., Han J. (2016). Post-Translational Modifications of Cardiac Mitochondrial Proteins in Cardiovascular Disease: Not Lost in Translation. Korean Circ. J..

[112] Nesci S., Trombetti F., Ventrella V., Pagliarani A. (2017). Post-translational modifications of the mitochondrial F1FO-ATPase. Biochim. Biophys. Acta Gen. Subj..

[113] Komander D., Rape M. (2012). The ubiquitin code. Annu. Rev. Biochem..

[114] Zimmermann M., Reichert A.S. (2017). How to get rid of mitochondria: Crosstalk and regulation of multiple mitophagy pathways. Biol. Chem..

[115] Desai S., Juncker M., Kim C. (2018). Regulation of mitophagy by the ubiquitin pathway in neurodegenerative diseases. Exp. Biol. Med..

[116] Harper J.W., Ordureau A., Heo J.M. (2018). Building and decoding ubiquitin chains for mitophagy. Nat. Rev. Mol. Cell Biol..

[117] Escobar-Henriques M., Langer T. (2014). Dynamic survey of mitochondria by ubiquitin. EMBO Rep..

[118] Ali S., McStay G.P. (2018). Regulation of Mitochondrial Dynamics by Proteolytic Processing and Protein Turnover. Antioxidants.

[119] Heaton S.M., Borg N.A., Dixit V.M. (2016). Ubiquitin in the activation and attenuation of innate antiviral immunity. J. Exp. Med..

[120] Liu J., Qian C., Cao X. (2016). Post-Translational Modification Control of Innate Immunity. Immunity.

[121] Johnson E.S. (2004). Protein modification by SUMO. Annu. Rev. Biochem..

[122] Enserink J.M. (2017). Regulation of Cellular Processes by SUMO: Understudied Topics. Adv. Exp. Med. Biol..

[123] Zhao X. (2018). SUMO-Mediated Regulation of Nuclear Functions and Signaling Processes. Mol. Cell.

[124] Harder Z., Zunino R., McBride H. (2004). Sumo1 conjugates mitochondrial substrates and participates in mitochondrial fission. Curr. Biol..

[125] Guo C., Hildick K.L., Luo J., Dearden L., Wilkinson K.A., Henley J.M. (2013). SENP3-mediated deSUMOylation of dynamin-related protein 1 promotes cell death following ischaemia. EMBO J..

[126] Prudent J., Zunino R., Sugiura A., Mattie S., Shore G.C., McBride H.M. (2015). MAPL SUMOylation of Drp1 Stabilizes an ER/Mitochondrial Platform Required for Cell Death. Mol. Cell.

[127] Choi S.G., Kim H., Jeong E.I., Lee H.J., Park S., Lee S.Y., Lee H.J., Lee S.W., Chung C.H., Jung Y.K. (2017). SUMO-Modified FADD Recruits Cytosolic Drp1 and Caspase-10 to Mitochondria for Regulated Necrosis. Mol. Cell. Biol..

[128] Guerra de Souza A.C., Prediger R.D., Cimarosti H. (2016). SUMO-regulated mitochondrial function in Parkinson’s disease. J. Neurochem..

[129] Im E., Yoo L., Hyun M., Shin W.H., Chung K.C. (2016). Covalent ISG15 conjugation positively regulates the ubiquitin E3 ligase activity of parkin. Open Biol..

[130] Yoshizumi T., Imamura H., Taku T., Kuroki T., Kawaguchi A., Ishikawa K., Nakada K., Koshiba T. (2017). RLR-mediated antiviral innate immunity requires oxidative phosphorylation activity. Sci. Rep..

[131] Berry B.J., Trewin A.J., Amitrano A.M., Kim M., Wojtovich A.P. (2018). Use the Protonmotive Force: Mitochondrial Uncoupling and Reactive Oxygen Species. J. Mol. Biol..

[132] He C., Carter A.B. (2015). The Metabolic Prospective and Redox Regulation of Macrophage Polarization. J. Clin. Cell. Immunol..

